# Serial changes in the head-shaft angle of proximal humeral fractures treated by placing locking plates: a retrospective study

**DOI:** 10.1186/s12891-018-2349-3

**Published:** 2018-11-30

**Authors:** Ji-Qi Wang, Bing-Jie Jiang, Wei-Jun Guo, You-Ming Zhao

**Affiliations:** Department of Orthopaedics, The Second Affiliated Hospital and Yuying Children’s Hospital of Wenzhou Medical University, 109# Xue Yuan Xi Road, Wenzhou, Zhejiang, 325000 China

**Keywords:** Proximal humeral fractures, Locking plate, Head-shaft angle, Functional outcomes, Medial support

## Abstract

**Background:**

Although the proximal humeral fractures (PHFs) treated with locking plate have been well applied, there are few studies concerning on the serial HSA changes after locking plate placement. The purpose of this retrospective study was to explored the clinical significance of serial HSA changes after surgery.

**Methods:**

We retrospectively analyzed the clinical data of 122 patients between January 2012 to December 2016 in our hospital. The serial change of the HSA and Neer’s score of 122 patients were recorded and analyzed. Then, we evaluated the HSA changes affected functional recovery in conjunction with medial support (MS). Moreover, multivariable linear regression analysis was performed to identify any potential confounding factors that may influence functional recovery.

**Results:**

Of 146 patients, 122 (50 males and 72 females) patients were finally enrolled in our study. Our preliminary data suggested that the most decrease of HSA occurred in the period of 1 to 3 months (*p* < 0.001) postoperatively, and functional recovery was significantly related with the change of HSA (R^2^ = 0.647, p < 0.001). The presence of MS plays an important role in maintaining postoperative HSA and restoring function. Moreover, Neer type 4 fracture, the difference between the postoperative HSA (on the injured side) and that of the uninjured side (the ΔHSA), and the HSA change to the end of follow-up were all significantly associated with functional recovery.

**Conclusions:**

Serial HSA changes were evident in PHF patients in whom locking plates had been inserted; it is essential to maintain reduction for 1–3 months postoperatively. MS is important in this context and surgeons must maximally restore MS. Furthermore, the functional outcome tended to improve when the HSA of the injured side was restored to a value close to that of the uninjured side.

## Background

Proximal humeral fractures (PHFs) represent 5% of all fractures [1]and require surgical treatment, especially in elderly patients for whom high-level functional recovery is important [2]. Placement of a proximal humeral internal locking system (PHILOS) is the current mainstay of treatment to achieve minimal loss of function [3]. Despite the advantages of this technique, the use of locking plates to surgically treat PHF is associated with high complication rates (16–36%) [4]. The major complications are reduction loss and varus malunion [3, 5, 6]; reduction loss can be associated with screw perforation of the articular surface, triggering a need for re-operation, or even causing complete fixation failure [7]. Recent studies showed that a lack of medial support (MS) might play an important role in reduction loss [8–10].

Generally, the humeral head-shaft angle (HSA) dictates the choice of plate length [11];our understanding of the HSA is continuously improving. To the best of our knowledge, the HSA is closely associated with shoulder function and is affected by many factors. However, only a few studies focused on serial HSA changes after locking plate placement. We explored the clinical significance of serial HSA changes after surgery. We retrospectively evaluated the changes, i.e. how they affected functional recovery in conjunction with MS, and the relationship between HSA changes and clinical outcomes after PHILOS fixation of PHFs.

## Methods

### Study design

We retrospectively reviewed serial HSA changes in PHF patients treated via locking plate placement. Our institutional ethics committee approved the study and all participants provided written informed consent prior to data collection.

### Patients

The inclusion criteria were as follows: an acute closed fracture < 14 days old treated via PHILOS; age ≥ 18 years; no complication such as nerve or vascular injury; no history of an upper extremity fracture; and complete follow-up. From January 2012 to December 2016, we treated 146 PHF patients using the PHILOS. Of these, 24 were excluded: 3 with open fractures, 3 with a history of upper extremity fracture, 4 with nerve injury, and 14 lost to follow-up. Thus, 122 patients (50 males and 72 females) met the inclusion criteria and were enrolled; we also measured the HSAs of the uninjured sides. According to the Neer classification [12], we treated 44 two-part, 58 three-part, and 20 four-part fracture patients.

### Surgical technique

All patients underwent a standard procedure via a deltopectoral approach while in the beach-chair position. After confirming satisfactory reduction, fragments were temporarily fixed with K-wires. Next, appropriate locking proximal humeral plates (Proximal Humeral Inter-locking System; Synthes; Oberdorf, Switzerland) were used to fix the fractures.

All patients underwent similar postoperative rehabilitation; the shoulders were sling-immobilised for 3–6 weeks, followed by controlled passive mobilisation exercises (pendulum exercise, flexion, and external rotation) for 1–6 weeks depending on fracture type and bone healing. For all patients, stretching and resistive strengthening exercises were recommended until complete fracture healing was evident on radiographs.

### Postoperative follow-up

**Fig. 1:**
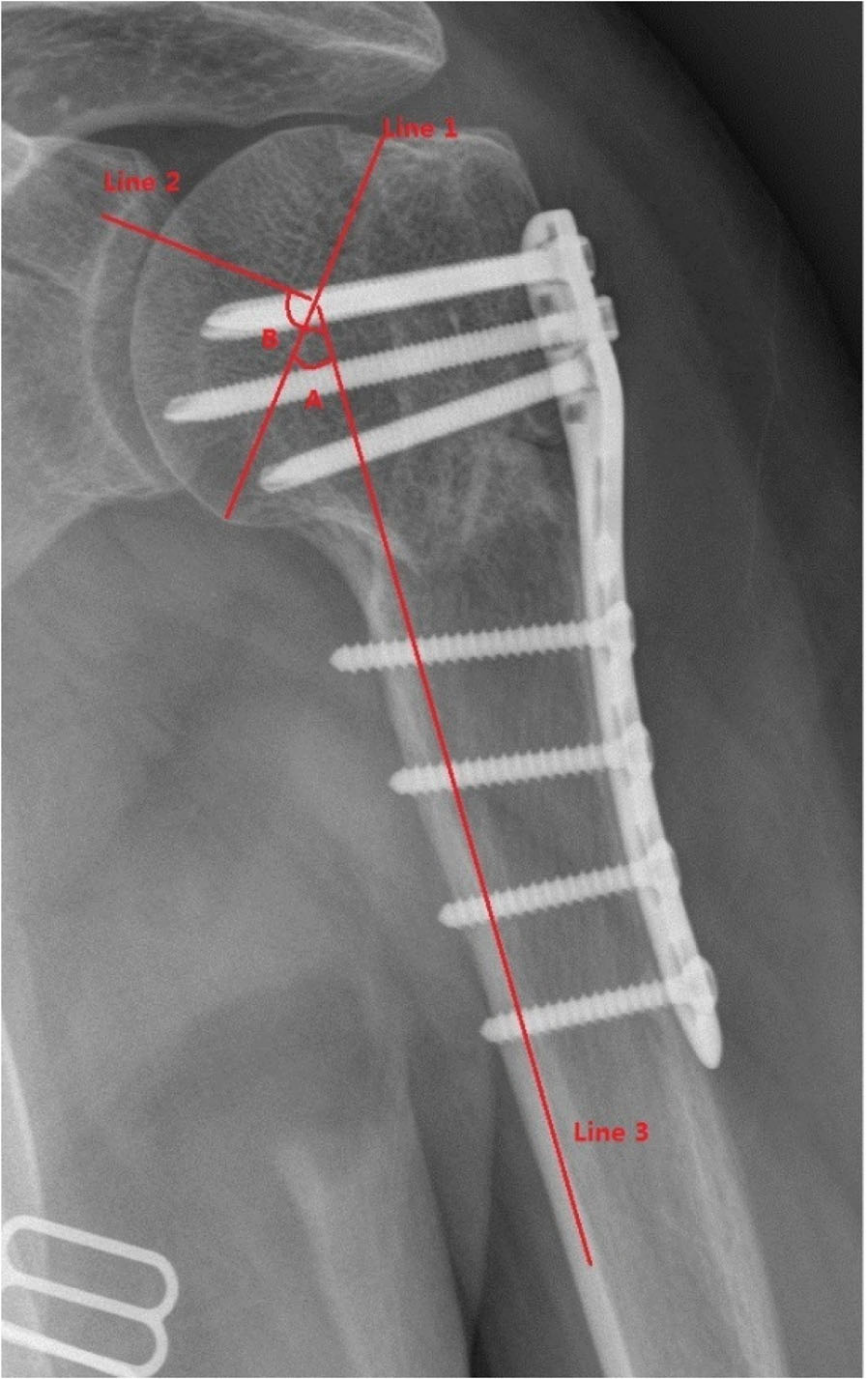
The head-shaft angle (HSA) measurement method. A vertical line is drawn from the superior to the inferior border of the articular surface (Line 1) and a line perpendicular to this line is then drawn to run through the centre of the humeral head (Line 2). The angle between the latter line and a line parallel to the long axis of the humeral shaft (Line 3) is the humeral head-shaft angle. We found that this angle (angle B) was equal to the angle between the vertical and the long axes (angle A) plus 90°

X-rays were taken immediately after the operation; at 1, 3, 6, and 12 months; and at the last follow-up. The HSA was measured as described by Agudelo et al. [13] (Fig. 1).A vertical line was first drawn from the superior to the inferior border of the articular surface (Line 1) and a line perpendicular to this line was then drawn through the centre of the humeral head (Line 2). The angle between the latter line and a line parallel to the long axis of the humeral shaft (Line 3) was defined as the humeral HSA. We found that this angle (angle B) was identical to the angle between the vertical and long axes (angle A), plus 90°. HSAs were independently assessed (twice) by two reviewers blinded to the clinical outcomes; the average of each set of four values was used in the analysis. In addition, following the recommendation of Greiner [14], an HSA < 120° was considered indicative of varus malunion. Using the criterion of Owsley [15], loss of reduction was defined as an HSA reduction > 10°. Functional recovery was evaluated using the Neer criteria [16] at the last follow-up.

### Statistical analysis

All statistical analyses were performed using SPSS software (ver. 19.0; SPSS Inc., Chicago, IL, USA). Numerical data were compared using Student’s t-test. One-factor analysis of variance (ANOVA) was employed to compare group means, and the LSD test was used to perform multiple comparisons. The Kruskal–Wallis test was employed to compare differences in Neer scores during follow-up; multiple comparisons were performed with the Tamhane T2 test. Categorical data were compared using the chi-squared (χ2) and Fisher’s exact tests. We performed linear regression analysis to identify relationships between functional outcomes and HSA changes. Univariate analysis was employed to explore associations between various factors and the Neer scores; multivariable linear regression was then performed to control for confounding effects. The level of significance was set to 0.05 for all analyses.

## Results

In total, 122 patients (50 males and 72 females) were included; their demographic data are listed in Table 1. The mean age was 61.1 years (range: 37–87 years). Over a mean of 16.2 months of follow-up (range: 12–24 months), all patients achieved bony union; the mean healing time was 4.4 months (range: 3–12 months). We treated 44 two-part, 58 three-part, and 20 four-part fracture patients; the mean HSA of the uninjured side was 136.5 ± 4.8°. Reduction loss was observed in 18 patients, of whom 16 experienced varus malunion, 2 subacromial impingement, 2 screw penetration requiring re-operations to remove the screws, and 4 delayed union that healed only after 12 months of follow-up.

**Table 1:** Patient’s demographic information

Total patients	122
Male	50
Female	72
Age (year)	61.1 ± 12.3
Fracture type	
two-part	44
three-part	58
four-part	20
Healing time (months)	4.4 ± 1.3
Follow-up time (months)	16.2 ± 3.2
Uninjured side HSA (°)	136.5 ± 4.8
Complications	
Reduction loss	18
Varus malunion	16
Subacromial impingement	2
Delayed union	4
Reoperation	2

*HSA* head-shaft angle

**Fig. 2:**
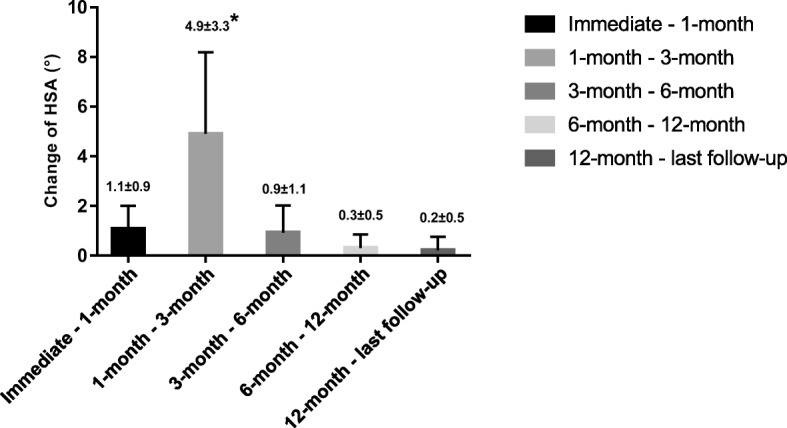
The change of HSA in each follow-up period. HSA, head-shaft angle; * indicate that most decrease of HSA occurs in the period of 1 to 3 months (*p* < 0.001)

**Fig. 3:**
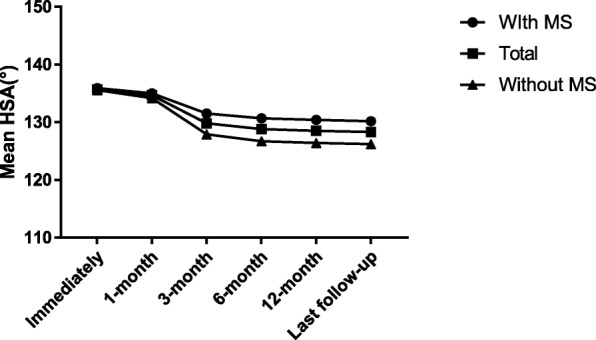
The HSA trends during follow-up. HSA, head-shaft angle; MS, medial support

**Fig. 4:**
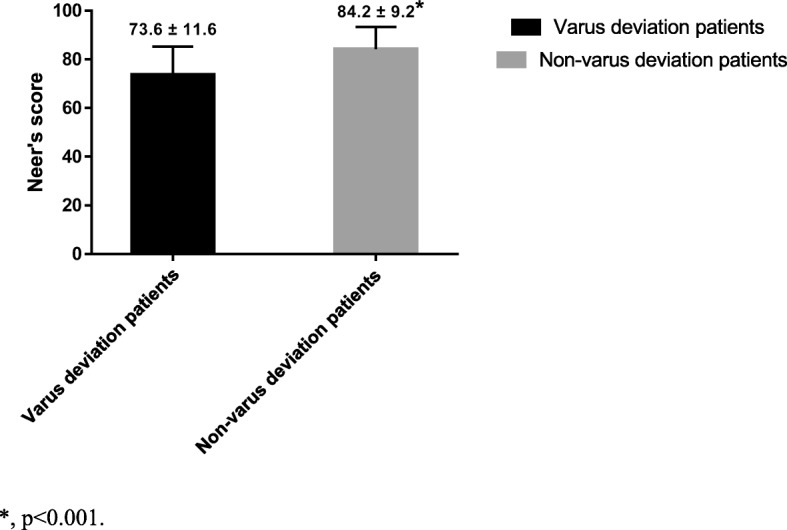
The mean Neer’s score in varus malunion and non-varus malunion patients. *, p < 0.001

We found that most of the HSA reduction occurred from 1 to 3 months postoperatively, and the reduction amount was 4.9 ± 3.3° (*p* < 0.001, Fig. 2). We divided the patients into two groups: with and without MS. We found no significant difference in HSA loss between the groups at 1 month postoperatively. However, after 3 months, the HSA of patients with MS was significantly greater than that of patients without MS (Table 2). Figure 3 shows the HSA trends during follow-up. The Neer score of patients with MS was significantly higher than that of patients lacking MS; the former patients enjoyed better functional recovery. Also, patients lacking MS were at a significantly increased risk of reduction loss than those with MS (Table 2, *p* = 0.005). The mean Neer score in the varus malunion group (HSA < 120°) at the last follow-up was 73.6 ± 11.6, versus 84.2 ± 9.2 in the non-varus malunion group (HSA ≥ 120°) (*p* < 0.001, Fig. 4).

**Table 2:** Comparison of head-shaft angle and clinical outcomes between patient with medial support and without medial support

		With MS	Without MS	*p* value
Mean HSA (°)	Immediate	135.6 ± 5.7	135.9 ± 5.0	0.752
	1-month	134.2 ± 5.8	135.0 ± 5.1	0.412
	3-month	127.9 ± 6.5	131.5 ± 6.7	0.003
	6-month	126.7 ± 6.3	130.7 ± 6.8	0.001
	12-month	126.4 ± 6.1	130.3 ± 6.7	< 0.001
	Last follow-up	126.2 ± 6.1	130.2 ± 6.9	0.001
Neer’s criteria				0.056
	Excellent (> 89)	14	28	
	Satisfactory (80–89)	20	24	
	Unsatisfactory (70–79)	14	12	
	Failure (< 70)	10	0	
Neer’s score		78.9 ± 11.6	86.3 ± 7.2	0.004
Complication				
	Reduction loss	14	4	0.005
	Varus malunion	10	6	0.199
	Subacromial impingement	0	2	0.175
	Delayed union	2	2	0.920
	Reoperation	2	0	0.134

*HSA* head-shaft angle, *MS* medial support

**Fig. 5:**
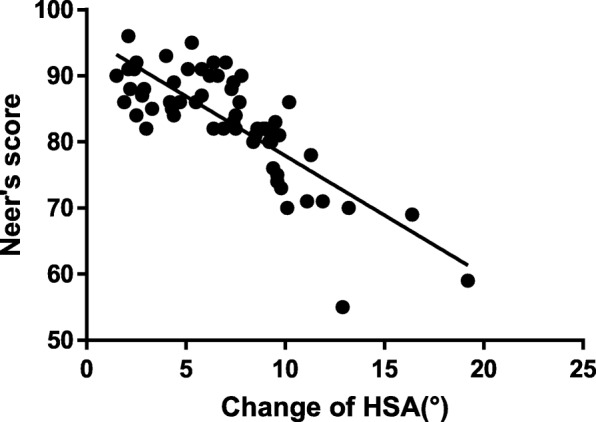
Linear regression analysis of Neer’s score and the HSA change to the end of follow-up. HSA, head-shaft angle

Linear regression showed that the Neer score was negatively associated with HSA change (R2 = 0.647, p < 0.001; Fig. 5). On univariate analyses, Neer type 4 fracture, the difference between the postoperative HSA (on the injured side) and that of the uninjured side (the ΔHSA), healing time, and the HSA change to the end of follow-up were all significantly associated with the Neer score (Table 3). On multivariate linear regression analysis, only healing time among the above four factors was excluded from the final model (R2 = 0.692, Table 4).

**Table 3:** Univariate association for each risk factor and Neer’s score

Characteristics	RR (95% CI)	p value
Age (year)	0.08 (−0.04, 0.19)	0.212
Sex		
Female	Ref.	
Male	0.25 (−2.72, 3.22)	0.868
Fracture type		
2	Ref.	
3	2.42 (0.69, 5.52)	0.129
4	4.21 (2.32, 8.40)	0.041
Uninjured side HSA	0.10 (−0.21, 0.40)	0.535
Postoperative HSA	0.17 (−0.11, 0.44)	0.245
ΔHSA	−2.04 (−3.36, −0.71)	0.003
Healing time (month)	−1.54 (−2.61, −0.47)	0.005
Change of HSA	−1.80 (−2.04, −1.56)	< 0.001

HSA, head-shaft angle; ΔHSA, the difference between the postoperative HSA (on the injured side) and that of the uninjured side; Change of HSA, the HSA change to the end of follow-up

**Table 4:** Multivariable linear regression model for Neer’s score

Characteristics	Coefficient	95% CI	p value
Fracture type 4	−4.31	−7.16, − 1.46	0.004
ΔHSA	−1.03	−1.84, −0.23	0.014
Healing time	0.08	−0.74, 0.89	0.857
Change of HSA	−1.70	−14.01, − 1.93	< 0.001

HSA, head-shaft angle; ΔHSA, the difference between the postoperative HSA (on the injured side) and that of the uninjured side; Change of HSA, the HSA change to the end of follow-up

## Discussion

HSA loss was maximal from 1 to 3 months postoperatively, in agreement with the literature [8, 17]. The average healing time was 4.4 months; the HSA remained essentially unchanged after 6 months. Of fractures that did not heal within 3 months, significant differences were evident between the HSA in the immediate postoperative phase and the values measured over 1–3 months. We suspected that this might reflect early voluntary exercise. Most patients could endure residual pain 1 month after surgery and, after 2 months of functional exercise, limb function partially recovered. Hence, some patients may have commenced internal forward rotation, and even stretching and weight-bearing activities. Muscle action during such exercises might change the HSA. In addition, the biomechanical stability of the proximal humerus may be compromised by fracture-healing during this period. Primitive bone callus formation varies among individuals; premature exercise may change the HSA but further research is required to confirm this.

The mean HSA of the with-MS group was significantly greater than that of the without-MS group at 3 months postoperatively (*p* = 0.003); MS was associated with less reduction loss. Overall, the reduction loss rate was significantly higher in the without-MS group (*p* = 0.005), consistent with other studies [18–20].Jung et al. [21] reported that inadequate MS was an independent risk factor for reduction loss after surgery to treat PHFs. In the absence of MS, the fixed-angle screws must serve as perpendicular struts aiding the humeral head to resist varus displacement. Therefore, MS is important for maintenance of reduction in patients with PHFs; surgeons must maximally restore MS.

Earlier studies on the functional and radiological outcomes of PHFs reached conflicting conclusions. Yüksel et al. [22] found no relationship between the HSA and the Constant score (which measures recovery); however, Bai et al. [23] reported that an HSA change > 10° significantly reduced the score. We investigated the relationship between the HSA and functional recovery in PHF patients in whom locking plates were placed and found that HSA change was negatively associated with recovery. Court-Brown et al. [24] found no association between increasing varus angulation and shoulder function; Capriccioso et al. [25] reported that the initial surgical neck displacement (varus or valgus) did not significantly affect functional outcomes. Conversely, 16 of our patients experienced varus malunion; their mean Neer score was 73.6 ± 11.6, thus lower than that of non-varus malunion patients (84.2 ± 9.2, *p* < 0.001). However, we would mention that three patients in the varus malunion group enjoyed excellent or satisfactory functional outcomes; all uninjured side HSAs were < 130°, and the Neer scores were 84, 92, and 83. A previous study found no significant between-group difference in bilateral HSAs [26].Therefore, we used multivariable linear regression to control for confounding effects; we found that Neer type 4 fracture, the postoperative HSA difference between the injured and uninjured side, and the change in HSA to completion of follow-up may influence the Neer score. Similarly, a previous study reported that, in patients with two- and three-part fractures, the overall functional results were usually satisfactory; but this was not always the case in those with four-part fractures [27, 28]. We suggest that the latter fractures usually develop in osteoporotic patients, often associated with proximal fractures and medial metaphyseal comminution [29].We found that the postoperative HSA difference between the treated and uninjured side, and the change in HSA to completion of follow-up, were negatively associated with the Neer score. Thus, restoration of the native HSA afforded better functional recovery. We identified a trend toward improved function when it was possible to restore the HSA of the injured side to a value similar to that of the uninjured side. Also, we could not rule out an effect of reduction loss in patients for whom the pre-injury HSA was < 130°.

Our study had certain limitations. First, it used a retrospective design and the sample size relatively small; we also cannot exclude potential effects of unmeasured factors. Furthermore, we prescribed a general postoperative rehabilitation protocol, but some patients may have individualised needs regarding the timing of passive range-of-motion exercises; this could affect the clinical and radiographic outcomes. A future prospective study with a larger sample size is required to validate our findings.

## Conclusions

Serial HSA changes were evident in PHF patients in whom locking plates had been inserted; it is essential to maintain reduction for 1–3 months postoperatively. MS is important in this context and surgeons must maximally restore MS. Furthermore, the functional outcome tended to improve when the HSA of the injured side was restored to a value close to that of the uninjured side.

## Abbreviations

HSA: head-shaft angle
MS: medial support
PHF: Proximal humeral fracture
PHILO: The proximal humeral internal locking system
ΔHSA: the difference between the postoperative HSA (on the injured side) and that of the uninjured side
